# Supplementary material for: Bioactive Ent-Kaurane Diterpenes Oridonin and Irudonin Prevent Cancer Cells Migration by Interacting with the Actin Cytoskeleton Controller Ezrin
Source: Int J Mol Sci. 2020 Sep 29;21(19):7186. doi: 10.3390/ijms21197186 (PMC7582544; doi:10.3390/ijms21197186)
Supplement: Supplementary file 1 [file ijms-21-07186-s001.zip › supproting/Figure S1.pdf]

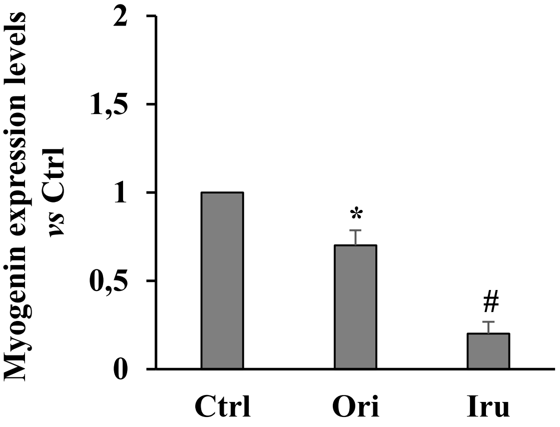


**Figure 2C**: Western blotting showing Myogenin protein expression levels. Fold change in Myogenin levels was calculated to un-treated control (cells cultured DMEM with 0.1% DMSO, vehicle) set as 1. #,* indicates significantly different from Ctrl (#p < 0.001 ;*p < 0.05).

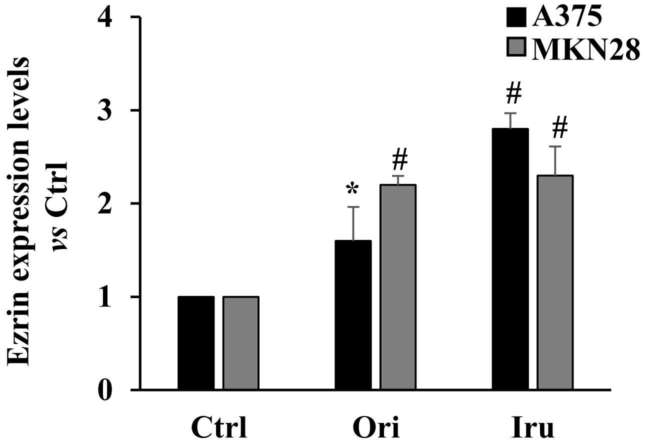


**Figure 4 A,B**: Western blotting showing Ezrin protein levels in A375 and MKN28 of DARTS experiments. Fold change in Ezrin levels was calculated relative to un-treated control set as 1. (*;#) indicate statistical significance toward control (*p < 0.05; #p < 0.001).

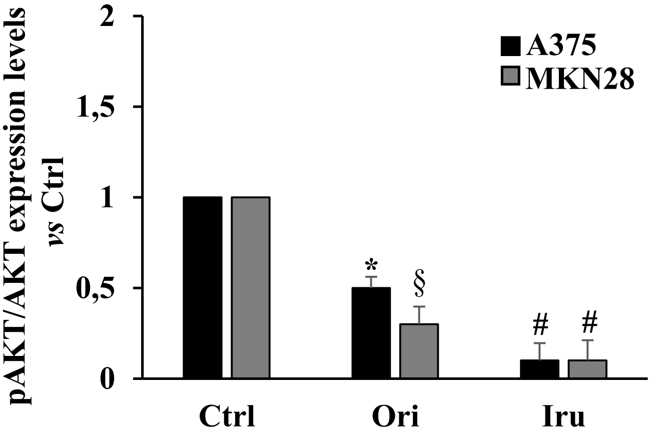


**Figure 5 A,B**: Western blotting analysis of phospho-AKT expression levels in (A) A375 and (B) MKN28 cells treated with Ori and Iru for 24 h. The relative fold change vs. untreated cells, set as 1, of protein levels is shown under each lane. Values marked with *, § or # were statistically different from control (*p < 0.05; §p < 0.01 , #p < 0.001).

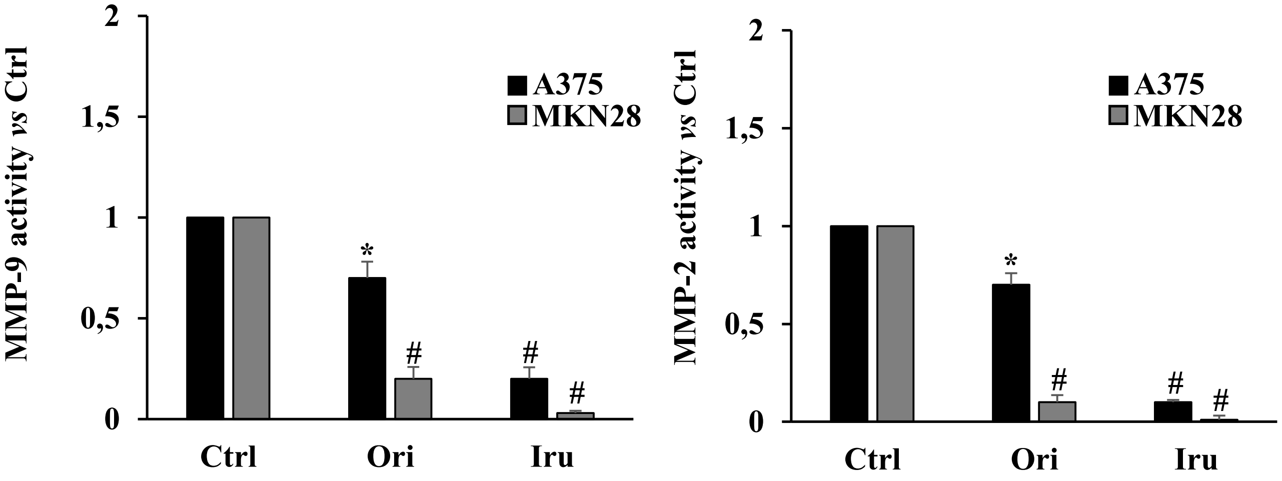


**Figure 6A**: Gelatin zymography of MMP-9 and MMP-2 in A375 and MKN-28 cells. Fold change in MMP-9 and MMP-2 activity was calculated by first normalizing with Ponceau-S staining of membranes (Control of protein loading) in individual samples and then relative to untreated control (cells cultured DMEM with 0.1% DMSO, vehicle) set as 1. The numbers with (*;#) were statistically different from control (*p < 0.05; #p < 0.001).

**Figure S1**: Histograms reporting the quantitative analyses and statistics of the blots showed in Figures 2, 4, 5 and 6. The numbers with (*;#) were statistically different from control (*p < 0.05; #p < 0.001).
