# Supplementary material for: Bioactive Ent-Kaurane Diterpenes Oridonin and Irudonin Prevent Cancer Cells Migration by Interacting with the Actin Cytoskeleton Controller Ezrin
Source: Int J Mol Sci. 2020 Sep 29;21(19):7186. doi: 10.3390/ijms21197186 (PMC7582544; doi:10.3390/ijms21197186)
Supplement: Supplementary file 1 [file ijms-21-07186-s001.zip › supproting/Figure S2.pdf]

A

Cell lines	Ori IC_{50}	Iru IC_{50}
C2C12	30	50
MKN28	20	25
A375	25	35

B

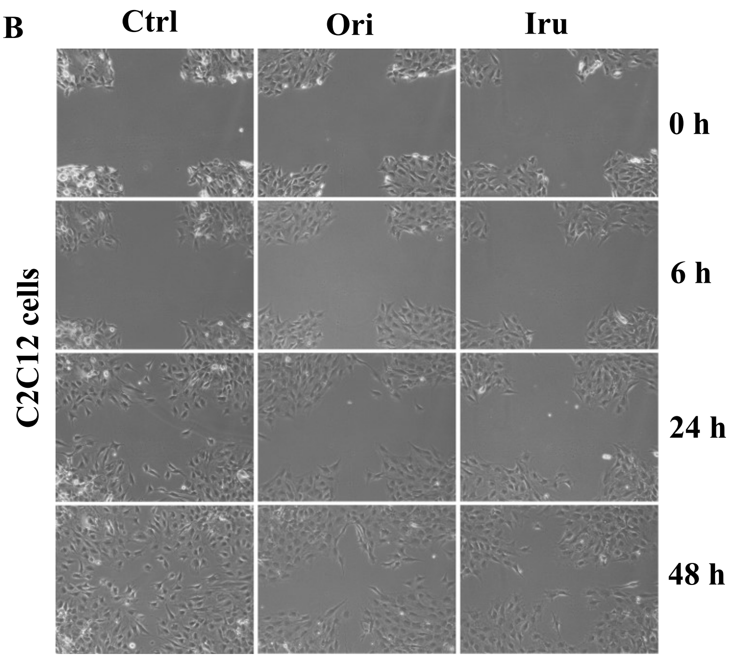

C

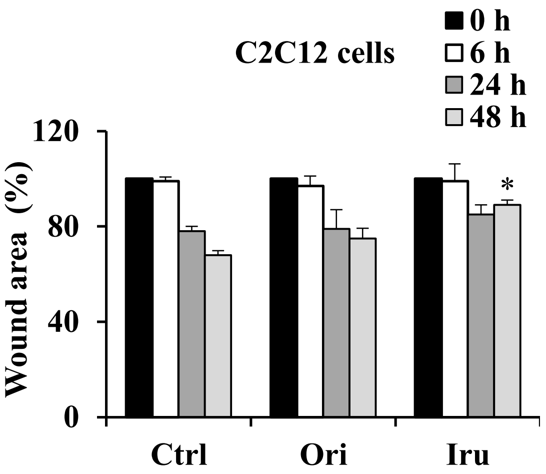

D

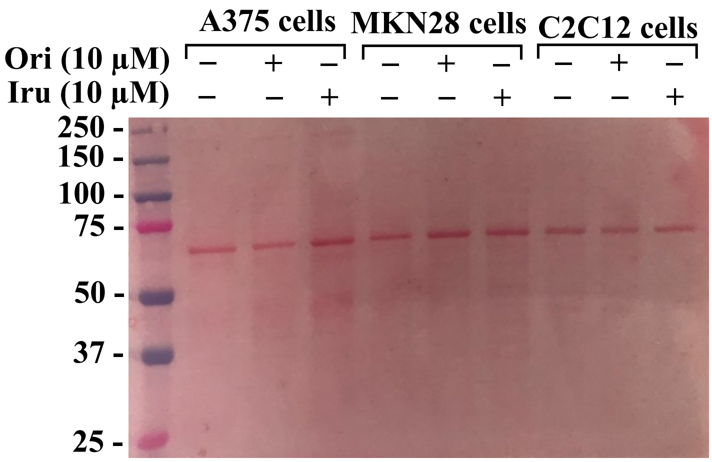

E

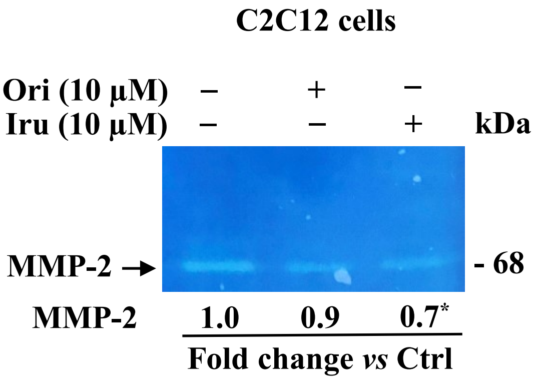

Figure S2: (A) IC50 values at 24 h, in C2C12, A375 and MKN28 cell lines. (B) Representative images of the wound healing assay of C2C12 cells. Cells were treated with 10 µM Ori or Iru and then captured at time 0, 6, 24 and 48h by a phase-contrast-microscope (10 × objective). (C) Quantification of wound area performed using the free image-processing software ImageJ, version 1.47. For each treatment, data show the wound area at the indicated time in comparison to that of the open wound at time 0, set as 100%. Results are presented as mean ± SD (n = 9). (D) Control of protein loading by Ponceau-S staining of membranes used for Gelatin Zymography carried out on cell condition media showed in Fig. 6A. (E) Gelatin zymography of MMP-9 and MMP-2 in C2C12 cells. Cell conditioned media were collected, concentrated by ultrafiltration and analyzed under non-reducing conditions through a 10 % SDS-polyacrylamide gel co-polymerized in the presence of gelatin (1 mg/ml). Fold change in MMP-2 activity was calculated by first normalizing with Ponceau-S staining of membranes (Control of protein loading) in individual samples and then relative to untreated control (cells cultured DMEM with 0.1% DMSO, vehicle) set as 1. The numbers with (*) were statistically different from control (*$p < 0.05$).
