# Supplementary material for: Bioactive Ent-Kaurane Diterpenes Oridonin and Irudonin Prevent Cancer Cells Migration by Interacting with the Actin Cytoskeleton Controller Ezrin
Source: Int J Mol Sci. 2020 Sep 29;21(19):7186. doi: 10.3390/ijms21197186 (PMC7582544; doi:10.3390/ijms21197186)
Supplement: Supplementary file 1 [file ijms-21-07186-s001.zip › supproting/Figure S3.pdf]

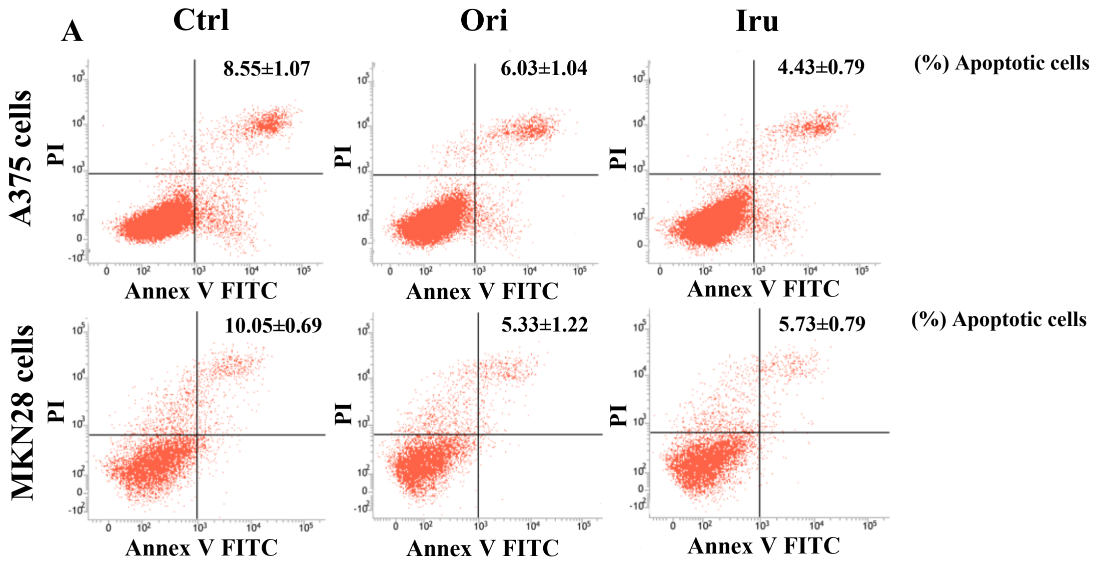

Figure S3 Apoptotic effect of Ori or Iru in **(A)** A375 and MKN28 cells. Cells were incubated for 24 h with 10 μM Ori or Iru. Annexin V–FITC binding and the propidium iodide incorporation were evaluated cytofluorimetrically. The rates of apoptosis were quantified (top right quadrant). The data are expressed as a percentage of apoptotic cells adding the percentual of Annexin V-positive/PI-negative cells (early apoptotic cells), and Annexin V-positive/PI-positive (late apoptotic cells). Data are shown from one of three independent experiments.
