# Supplementary material for: Bioactive Ent-Kaurane Diterpenes Oridonin and Irudonin Prevent Cancer Cells Migration by Interacting with the Actin Cytoskeleton Controller Ezrin
Source: Int J Mol Sci. 2020 Sep 29;21(19):7186. doi: 10.3390/ijms21197186 (PMC7582544; doi:10.3390/ijms21197186)
Supplement: Supplementary file 1 [file ijms-21-07186-s001.zip › supproting/Figure S4.pdf]

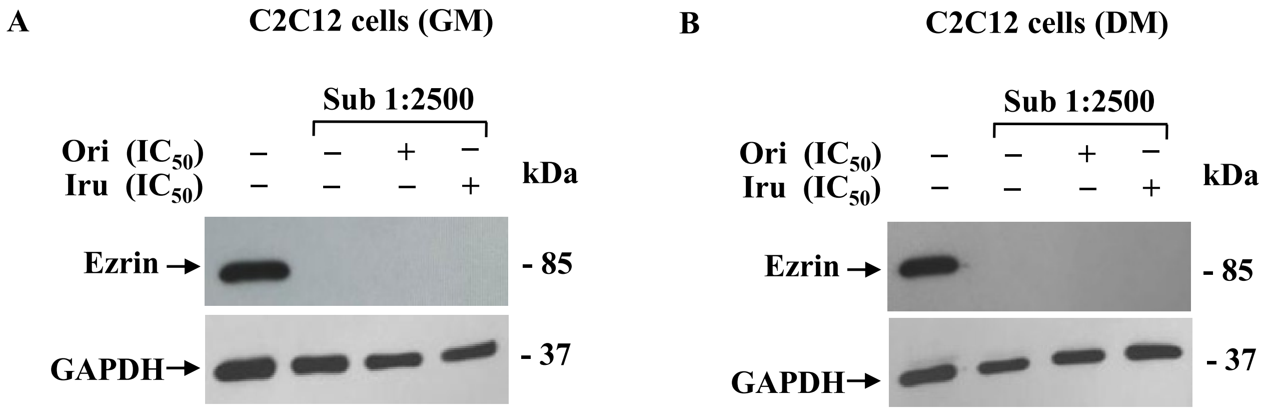

Figure S4: Western blotting showing Ezrin protein levels in (A) C2C12 undifferentiated cells (cultured in GM) and (B) C2C12 differentiated cells (cultured in DM) treated with IC50 values of Ori or Iru for 2h. After treatments, cells were lysated and the protein extracts were subjected to a limited digestion with subtilisin (1:2500). The resulting partially hydrolyzed protein mixtures were used for Western blotting analysis of DARTS experiments. GAPDH was used as a control protein as it partially resists to subtilisin catalyzed proteolysis.
