# Supplementary material for: Bioactive Ent-Kaurane Diterpenes Oridonin and Irudonin Prevent Cancer Cells Migration by Interacting with the Actin Cytoskeleton Controller Ezrin
Source: Int J Mol Sci. 2020 Sep 29;21(19):7186. doi: 10.3390/ijms21197186 (PMC7582544; doi:10.3390/ijms21197186)
Supplement: Supplementary file 1 [file ijms-21-07186-s001.zip › supproting/Figure S5.pdf]

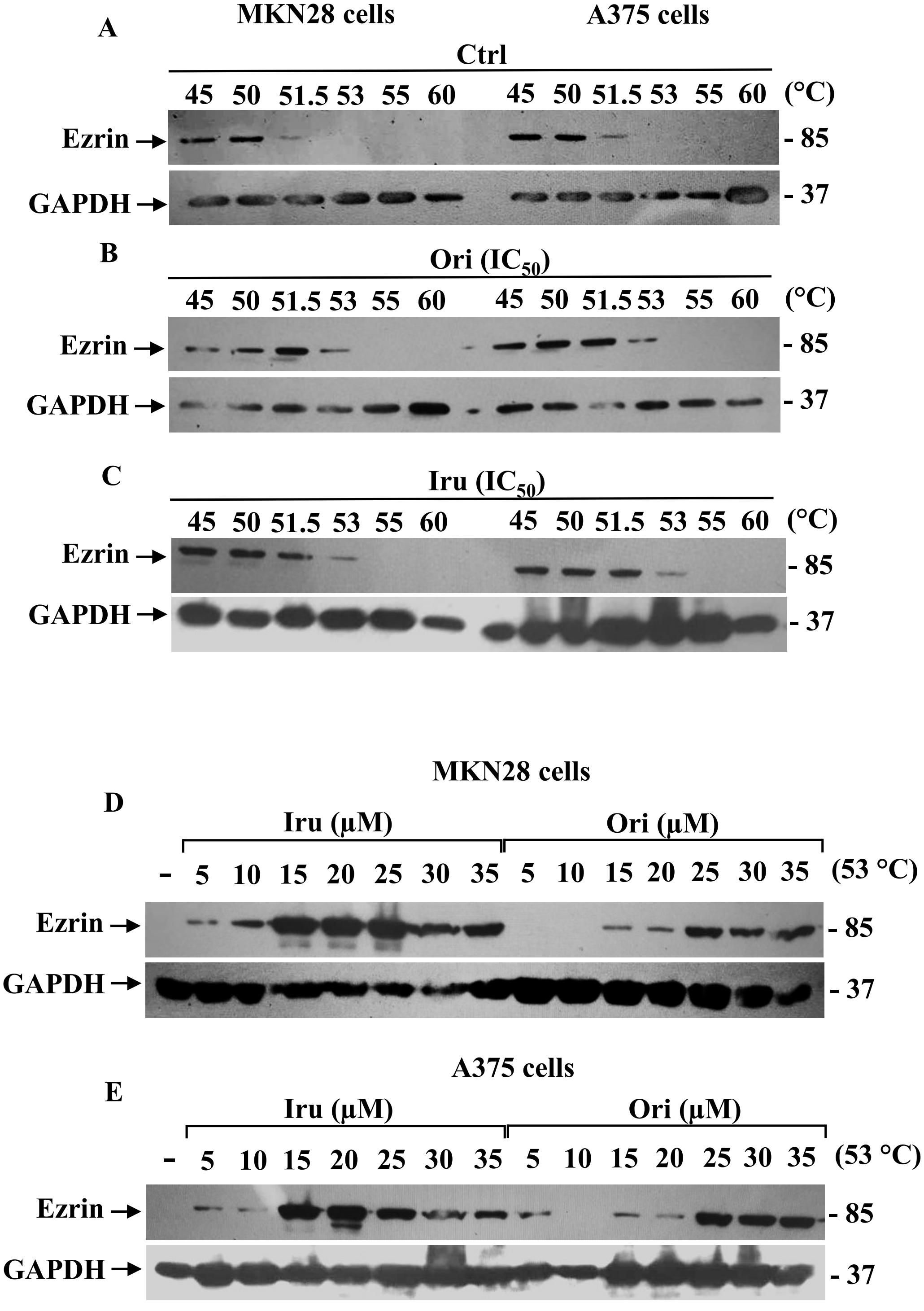

Figure S5: Representative images of CETSA Western blotting, showing Ezrin protein levels in A375 and MKN28 cells. The Ezrin Tm was evaluated in untreated cells (A), or following a 2 h exposure to a concentration of Ori (B) or Iru (C) corresponding to the respective IC $_{50}$. EC50 of each compound was estimated by performing CETSA experiments after incubation with different amounts of or Iru of MKN28 (D) or A375 (E) cells.
